# Supplementary material for: Blood biomarkers of Alzheimer’s disease and progression across different stages of cognitive decline in the community
Source: Nat Commun. 2025 Nov 23;16:10412. doi: 10.1038/s41467-025-66728-2 (PMC12644782; doi:10.1038/s41467-025-66728-2)
Supplement: Supplementary file 1 — Supplementary Information [file 41467_2025_66728_MOESM1_ESM.pdf]

Supplementary material

Supplementary Table S1. Levels of blood biomarkers of Alzheimer's disease (AD) and hazard ratio (HR) of progression from normal cognition (NC) to mild cognitive impairment (MCI), reversion from MCI to NC, and progression from MCI to AD dementia.

	**From NC to MCI**			**From MCI to NC**			**From MCI to AD dementia**		
	N transitions/ participants	HR (95%CI) Basic model	HR (95%CI) Full adj.	N transitions/ participants	HR (95%CI) Basic model	HR (95%CI) Full adj.	N transitions/ participants	HR (95%CI) Basic model	HR (95%CI) Full adj.
**Aß42/40**									
Low vs High	138/1119 vs 173/1029	0.88 (0.66, 1.17)	0.85 (0.63, 1.15)	129/1119 vs 164/1029	**0.66 (0.47, 0.92)**	**0.65 (0.46, 0.94)**	145/1119 vs 67/1029	**1.40 (1.08, 1.81)**	**1.30 (1.01, 1.67)**
**P-tau181**									
High vs Low	89/781 vs 222/1367	1.00 (0.73, 1.38)	1.03 (0.74, 1.43)	77/781 vs 216/1367	**0.59 (0.40, 0.87)**	**0.61 (0.40, 0.93)**	128/781 vs 84/1367	**1.52 (1.17, 1.96)**	**1.36 (1.05, 1.76)**
**P-tau217**									
High vs Low	81/803 vs 230/1345	1.19 (0.87, 1.63)	1.23 (0.89, 1.71)	72/803 vs 221/1345	0.73 (0.49, 1.09)	0.78 (0.50, 1.20)	140/803 vs 72/1345	**2.11 (1.61, 2.76)**	**1.87 (1.43, 2.46)**
**T-tau**									
High vs Low	152/1090 vs 159/1058	1.10 (0.82, 1.46)	1.00 (0.74, 1.34)	133/1090 vs 160/1058	0.98 (0.69, 1.38)	0.89 (0.62, 1.29)	132/1090 vs 80/1058	**1.45 (1.13, 1.87)**	**1.35 (1.05, 1.73)**
**NfL**									
High vs Low	97/941 vs 214/1207	1.15 (0.82, 1.60)	1.05 (0.69, 1.59)	84/941 vs 209/1207	0.69 (0.46, 1.02)	**0.58 (0.35, 0.97)**	156/941 vs 56/1207	**2.34 (1.77, 3.11)**	**2.01 (1.50, 2.70)**
**GFAP**									
High vs Low	99/879 vs 212/1269	1.12 (0.82, 1.54)	1.14 (0.81, 1.60)	87/879 vs 206/1269	**0.67 (0.46, 0.97)**	0.66 (0.43, 1.01)	154/879 vs 58/1269	**1.88 (1.42, 2.49)**	**1.66 (1.25, 2.20)**

Hazard Ratios (HR) with 95% Confidence Intervals (CI) are derived from multistate Markov models, using age as time scale. The basic model is adjusted for sex and education; the fully adjusted model is further adjusted for chronic kidney disease, heart diseases, cerebrovascular disease, anemia and obesity. Cut-offs: 0.057 for Aß-42/40 ratio, 1.512 pg/mL for p-tau181, 0.134 pg/mL for p-tau217, 0.832 pg/mL for t-tau, 20.171 pg/mL for NfL and 142.515 pg/mL for GFAP. Abbreviations: Aß42/40: amyloid beta 42/40; MCI: mild cognitive impairment; NC: normal cognition; p-tau181: phosphorylated tau 181; p-tau217: phosphorylated tau 217; t-tau: total tau; NfL: neurofilament light chain; GFAP: glial fibrillary acidic protein.

Supplementary Table S2. Levels of blood biomarkers of Alzheimer's disease and hazard ratio (HR) of progression from normal cognition (NC) to mild cognitive impairment (MCI), reversion from MCI to NC, and progression from MCI to all-cause dementia, for participants below or above 78 years old.

	**From NC to MCI**		**From MCI to NC**		**From MCI to all-cause dementia**	
	<78 years old HR (95%CI)	≥78 years old HR (95%CI)	<78 years old HR (95%CI)	≥78 years old HR (95%CI)	<78 years old HR (95%CI)	≥78 years old HR (95%CI)
**Aß-42/40**						
Low vs High	0.78 (0.52, 1.17)	1.35 (0.65, 2.82)	0.64 (0.39, 1.04)	1.15 (0.38, 3.51)	**1.53 (1.04, 2.25)**	1.28 (0.96, 1.70)
**P-tau181**						
High vs Low	0.74 (0.47, 1.16)	1.27 (0.75, 2.18)	**0.41 (0.23, 0.73)**	0.89 (0.40, 1.99)	**1.51 (1.00, 2.30)**	**1.40 (1.08, 1.82)**
**P-tau217**						
High vs Low	1.03 (0.63, 1.70)	1.17 (0.72, 1.90)	0.66 (0.33, 1.33)	0.77 (0.37, 1.58)	**2.08 (1.35, 3.22)**	**1.68 (1.29, 2.19)**
**T-tau**						
High vs Low	1.10 (0.72, 1.69)	1.39 (0.79, 2.44)	0.99 (0.59, 1.66)	1.44 (0.62, 3.35)	**1.65 (1.12, 2.42)**	**1.41 (1.09, 1.82)**
**NfL**						
High vs Low	1.41 (0.73, 2.76)	**0.38 (0.17, 0.85)**	0.68 (0.27, 1.70)	**0.24 (0.10, 0.61)**	**2.47 (1.66, 3.68)**	**1.60 (1.20, 2.12)**
**GFAP**						
High vs Low	1.18 (0.69, 2.01)	0.74 (0.44, 1.25)	0.58 (0.28, 1.19)	**0.46 (0.23, 0.92)**	**1.73 (1.15, 2.60)**	**1.41 (1.08, 1.84)**

Hazard Ratios (HR) with 95% Confidence Intervals (CI) are derived from multistate Markov models, using age as time scale and adjusted for sex and education. Cut-offs: 0.057 for Aß-42/40 ratio, 1.512 pg/mL for p-tau181, 0.134 pg/mL for p-tau217, 0.832 pg/mL for t-tau, 20.171 pg/mL for NfL and 142.515 pg/mL for GFAP. Abbreviations: Aß42/40: amyloid beta 42/40; MCI: mild cognitive impairment; NC: normal cognition; p-tau181: phosphorylated tau 181; p-tau217: phosphorylated tau 217; t-tau: total tau; NfL: neurofilament light chain; GFAP: glial fibrillary acidic protein.

Supplementary Table S3. Levels of blood biomarkers of Alzheimer's disease (AD) and hazard ratio (HR) of progression from normal cognition (NC) to mild cognitive impairment (MCI), reversion from MCI to NC, and progression from MCI to AD dementia, for participants below or above 78 years old.

	**From NC to MCI**		**From MCI to NC**		**From MCI to AD dementia**	
	<78 years old HR (95%CI)	≥78 years old HR (95%CI)	<78 years old HR (95%CI)	≥78 years old HR (95%CI)	<78 years old HR (95%CI)	≥78 years old HR (95%CI)
**Aß-42/40**						
Low vs High	0.70 (0.47, 1.05)	1.05 (0.73, 1.52)	0.56 (0.36, 0.89)	0.74 (0.47, 1.15)	1.51 (0.95, 2.38)	**1.36 (1.03, 1.80)**
**P-tau181**						
High vs Low	0.79 (0.51, 1.24)	1.17 (0.80, 1.71)	**0.42 (0.24, 0.74)**	0.73 (0.45, 1.17)	**1.69 (1.04, 2.74)**	**1.50 (1.13, 1.97)**
**P-tau217**						
High vs Low	1.17 (0.70, 1.96)	1.23 (0.83, 1.81)	0.75 (0.37, 1.51)	0.74 (0.46, 1.20)	**2.48 (1.48, 4.18)**	**2.04 (1.53, 2.73)**
**T-tau**						
High vs Low	1.05 (0.70, 1.57)	1.15 (0.80, 1.65)	0.92 (0.58, 1.46)	1.04 (0.66, 1.66)	**1.76 (1.10, 2.80)**	**1.37 (1.04, 1.81)**
**NfL**						
High vs Low	**2.08 (1.06, 4.09)**	0.80 (0.52, 1.23)	0.95 (0.35, 2.55)	**0.61 (0.37, 1.00)**	**3.06 (1.96, 4.78)**	**2.06 (1.50, 2.82)**
**GFAP**						
High vs Low	1.24 (0.78, 1.99)	1.04 (0.71, 1.53)	0.59 (0.32, 1.10)	0.71 (0.45, 1.13)	**2.29 (1.42, 3.70)**	**1.79 (1.31, 2.44)**

Hazard Ratios (HR) with 95% Confidence Intervals (CI) are derived from multistate Markov models, using age as time scale and adjusted for sex and education. Cut-offs: 0.057 for Aß-42/40 ratio, 1.512 pg/mL for p-tau181, 0.134 pg/mL for p-tau217, 0.832 pg/mL for t-tau, 20.171 pg/mL for NfL and 142.515 pg/mL for GFAP. Abbreviations: Aß42/40: amyloid beta 42/40; MCI: mild cognitive impairment; NC: normal cognition; p-tau181: phosphorylated tau 181; p-tau217: phosphorylated tau 217; t-tau: total tau; NfL: neurofilament light chain; GFAP: glial fibrillary acidic protein.

Supplementary Table S4. Levels of blood biomarkers of Alzheimer's disease and hazard ratio (HR) of progression from normal cognition (NC) to mild cognitive impairment (MCI), reversion from MCI to NC, and progression from MCI to all-cause dementia, stratified by sex.

	**From NC to MCI**		**From MCI to NC**		**From MCI to all-cause dementia**	
	Men HR (95%CI)	Women HR (95%CI)	Men HR (95%CI)	Women HR (95%CI)	Men HR (95%CI)	Women HR (95%CI)
**Aß-42/40**						
Low vs High	0.62 (0.31, 1.22)	1.08 (0.73, 1.60)	0.47 (0.20, 1.12)	0.84 (0.50, 1.41)	1.26 (0.84, 1.88)	1.28 (0.98, 1.68)
**P-tau181**						
High vs Low	0.73 (0.35, 1.52)	1.01 (0.61, 1.68)	0.49 (0.19, 1.28)	0.58 (0.29, 1.14)	1.14 (0.76, 1.73)	**1.52 (1.15, 2.01)**
**P-tau217**						
High vs Low	0.87 (0.42, 1.79)	1.18 (0.74, 1.88)	0.66 (0.24, 1.82)	0.70 (0.37, 1.32)	**1.55 (1.00, 2.39)**	**1.89 (1.43, 2.52)**
**T-tau**						
High vs Low	1.40 (0.75, 2.62)	1.50 (0.83, 2.71)	2.37 (0.97, 5.63)	1.24 (0.55, 2.82)	**1.53 (1.03, 2.28)**	**1.43 (1.08, 1.88)**
**NfL**						
High vs Low	1.36 (0.66, 2.80)	0.77 (0.30, 2.01)	0.86 (0.33, 2.24)	0.50 (0.16, 1.56)	**1.82 (1.18, 2.82)**	**1.97 (1.41, 2.73)**
**GFAP**						
High vs Low	0.88 (0.46, 1.69)	0.89 (0.54, 1.48)	**0.32 (0.12, 0.82)**	0.62 (0.33, 1.15)	1.24 (0.80, 1.92)	**1.64 (1.23, 2.20)**

Hazard Ratios (HR) with 95% Confidence Intervals (CI) are derived from multistate Markov models, using age as time scale and adjusted for education. Cut-offs: 0.057 for Aß-42/40 ratio, 1.512 pg/mL for p-tau181, 0.134 pg/mL for p-tau217, 0.832 pg/mL for t-tau, 20.171 pg/mL for NfL and 142.515 pg/mL for GFAP. Abbreviations: Aß42/40: amyloid beta 42/40; MCI: mild cognitive impairment; NC: normal cognition; p-tau181: phosphorylated tau 181; p-tau217: phosphorylated tau 217; t-tau: total tau; NfL: neurofilament light chain; GFAP: glial fibrillary acidic protein.

Supplementary Table S5. Levels of blood biomarkers of Alzheimer's disease (AD) and hazard ratio (HR) of progression from normal cognition (NC) to mild cognitive impairment (MCI), reversion from MCI to NC, and progression from MCI to AD dementia, stratified by sex.

	**From NC to MCI**		**From MCI to NC**		**From MCI to AD dementia**	
	Men HR (95%CI)	Women HR (95%CI)	Men HR (95%CI)	Women HR (95%CI)	Men HR (95%CI)	Women HR (95%CI)
**Aß-42/40**						
Low vs High	0.81 (0.52, 1.27)	0.90 (0.62, 1.33)	0.58 (0.32, 1.05)	0.68 (0.44, 1.06)	1.49 (0.91, 2.43)	1.35 (0.98, 1.86)
**P-tau181**						
High vs Low	1.07 (0.65, 1.76)	0.98 (0.65, 1.48)	0.69 (0.36, 1.33)	**0.55 (0.33, 0.92)**	1.45 (0.89, 2.36)	**1.62 (1.18, 2.22)**
**P-tau217**						
High vs Low	1.19 (0.72, 1.96)	1.17 (0.78, 1.76)	0.85 (0.42, 1.73)	0.66 (0.40, 1.10)	**2.41 (1.38, 4.20)**	**2.13 (1.55, 2.94)**
**T-tau**						
High vs Low	1.06 (0.68, 1.66)	1.23 (0.83, 1.82)	1.35 (0.74, 2.48)	0.97 (0.60, 1.57)	1.41 (0.88, 2.26)	**1.50 (1.09, 2.06)**
**NfL**						
High vs Low	**1.89 (1.13, 3.17)**	0.83 (0.48, 1.43)	0.89 (0.45, 1.76)	0.54 (0.29, 1.03)	**2.33 (1.42, 3.83)**	**2.44 (1.70, 3.49)**
**GFAP**						
High vs Low	1.32 (0.82, 2.12)	0.99 (0.65, 1.52)	**0.46 (0.22, 0.93)**	0.71 (0.45, 1.15)	**2.01 (1.18, 3.45)**	**1.98 (1.39, 2.81)**

Hazard Ratios (HR) with 95% Confidence Intervals (CI) are derived from multistate Markov models, using age as time scale and adjusted for education. Cut-offs: 0.057 for Aß-42/40 ratio, 1.512 pg/mL for p-tau181, 0.134 pg/mL for p-tau217, 0.832 pg/mL for t-tau, 20.171 pg/mL for NfL and 142.515 pg/mL for GFAP. Abbreviations: Aß42/40: amyloid beta 42/40; MCI: mild cognitive impairment; NC: normal cognition; p-tau181: phosphorylated tau 181; p-tau217: phosphorylated tau 217; t-tau: total tau; NfL: neurofilament light chain; GFAP: glial fibrillary acidic protein.

Supplementary Table S6. Levels of blood biomarkers of Alzheimer's disease and hazard ratio (HR) of progression from normal cognition (NC) to mild cognitive impairment (MCI), reversion from MCI to NC, and progression from MCI to all-cause dementia, excluding participants with MCI at baseline (n= 381).

	**From NC to MCI**		**From MCI to NC**		**From MCI to all-cause dementia**	
	N transitions/ participants	HR (95%CI)	N transitions/ participants	HR (95%CI)	N transitions/ participants	HR (95%CI)
**Aß-42/40**						
Low vs High	122/908 vs 153/859	0.69 (0.46, 1.06)	36/908 vs 71/859	**0.52 (0.27, 1.00)**	180/908 vs 95/859	**1.35 (1.01, 1.80)**
**P-tau181**						
High vs Low	82/637 vs 193/1130	1.15 (0.71, 1.87)	30/637 vs 77/1130	0.77 (0.35, 1.69)	163/637 vs 112/1130	1.31 (0.97, 1.76)
**P-tau217**						
High vs Low	74/657 vs 201/1110	1.09 (0.70, 1.71)	27/657 vs 80/1110	0.82 (0.37, 1.79)	173/657 vs 102/1110	**1.76 (1.29, 2.40)**
**T-tau**						
High vs Low	137/906 vs 138/861	1.13 (0.75, 1.69)	49/906 vs 58/861	1.06 (0.55, 2.05)	175/906 vs 100/861	**1.46 (1.10, 1.94)**
**NfL**						
High vs Low	90/780 vs 185/987	1.02 (0.68, 1.55)	34/780 vs 73/987	0.58 (0.31, 1.10)	202/780 vs 73/987	**1.59 (1.16, 2.18)**
**GFAP**						
High vs Low	87/715 vs 188/1052	0.88 (0.58, 1.35)	29/715 vs 78/1052	0.54 (0.27, 1.06)	192/715 vs 83/1052	**1.45 (1.06, 1.98)**

Hazard Ratios (HR) with 95% Confidence Intervals (CI) are derived from multistate Markov models, using age as time scale and adjusted for education. Cut-offs: 0.057 for Aß-42/40 ratio, 1.512 pg/mL for p-tau181, 0.134 pg/mL for p-tau217, 0.832 pg/mL for t-tau, 20.171 pg/mL for NfL and 142.515 pg/mL for GFAP. Abbreviations: Aß42/40: amyloid beta 42/40; MCI: mild cognitive impairment; NC: normal cognition; p-tau181: phosphorylated tau 181; p-tau217: phosphorylated tau 217; t-tau: total tau; NfL: neurofilament light chain; GFAP: glial fibrillary acidic protein.

Supplementary Table S7. Levels of blood biomarkers of Alzheimer's disease (AD) and hazard ratio (HR) of progression from normal cognition (NC) to mild cognitive impairment (MCI), reversion from MCI to NC, and progression from MCI to AD dementia, excluding participants with MCI at baseline (n= 381).

	**From NC to MCI**		**From MCI to NC**		**From MCI to AD dementia**	
	N transitions/ participants	HR (95%CI)	N transitions/ participants	HR (95%CI)	N transitions/ participants	HR (95%CI)
**Aß-42/40**						
Low vs High	122/908 vs 153/859	0.74 (0.53, 1.04)	36/908 vs 71/859	**0.47 (0.27, 0.84)**	108/908 vs 46/859	**1.59 (1.11, 2.27)**
**P-tau181**						
High vs Low	82/637 vs 193/1130	1.11 (0.78, 1.58)	30/637 vs 77/1130	0.60 (0.32, 1.14)	91/637 vs 63/1130	1.41 (0.98, 2.03)
**P-tau217**						
High vs Low	74/657 vs 201/1110	1.22 (0.87, 1.72)	27/657 vs 80/1110	0.77 (0.41, 1.45)	102/657 vs 52/1110	**2.33 (1.63, 3.33)**
**T-tau**						
High vs Low	137/906 vs 138/861	1.10 (0.79, 1.53)	49/906 vs 58/861	0.97 (0.55, 1.73)	102/906 vs 52/861	**1.70 (1.19, 2.41)**
**NfL**						
High vs Low	90/780 vs 185/987	1.17 (0.81, 1.69)	34/780 vs 73/987	**0.54 (0.31, 0.97)**	115/780 vs 39/987	**2.10 (1.41, 3.14)**
**GFAP**						
High vs Low	87/715 vs 188/1052	1.08 (0.77, 1.50)	29/715 vs 78/1052	0.60 (0.33, 1.07)	109/715 vs 45/1052	**1.94 (1.32, 2.83)**

Hazard Ratios (HR) with 95% Confidence Intervals (CI) are derived from multistate Markov models, using age as time scale and adjusted for education. Cut-offs: 0.057 for Aß-42/40 ratio, 1.512 pg/mL for p-tau181, 0.134 pg/mL for p-tau217, 0.832 pg/mL for t-tau, 20.171 pg/mL for NfL and 142.515 pg/mL for GFAP. Abbreviations: Aß42/40: amyloid beta 42/40; MCI: mild cognitive impairment; NC: normal cognition; p-tau181: phosphorylated tau 181; p-tau217: phosphorylated tau 217; t-tau: total tau; NfL: neurofilament light chain; GFAP: glial fibrillary acidic protein.

Supplementary Table S8. Levels of blood biomarkers of Alzheimer's disease (AD) and hazard ratio (HR) of progression from normal cognition (NC) to mild cognitive impairment (MCI), reversion from MCI to NC, and progression from MCI to all-cause dementia, applying inverse probability weighting (IPW).

	**From NC to MCI**	**From MCI to NC**	**From MCI to all-cause dementia**
	HR (95%CI)	HR (95%CI)	HR (95%CI)
**Aß-42/40**			
Low vs High	0.96 (0.71, 1.30)	0.75 (0.50, 1.11)	**1.30 (1.05, 1.60)**
**P-tau181**			
High vs Low	0.92 (0.64, 1.32)	**0.55 (0.34, 0.90)**	**1.37 (1.10, 1.70)**
**P-tau217**			
High vs Low	1.07 (0.75, 1.53)	0.69 (0.41, 1.14)	**1.75 (1.39, 2.19)**
**T-tau**			
High vs Low	1.17 (0.85, 1.63)	1.10 (0.70, 1.72)	**1.42 (1.15, 1.75)**
**NfL**			
High vs Low	0.98 (0.65, 1.50)	0.62 (0.37, 1.05)	**1.90 (1.49, 2.42)**
**GFAP**			
High vs Low	0.92 (0.65, 1.30)	**0.55 (0.35, 0.86)**	**1.49 (1.18, 1.87)**

Hazard Ratios (HR) with 95% Confidence Intervals (CI) are derived from multistate Markov models, using age as time scale and adjusted for sex and education. Weights were derived from logistic regression models including age, sex, education, number of chronic diseases, and biomarker levels. Cut-offs: 0.057 for Aß-42/40 ratio, 1.512 pg/mL for p-tau181, 0.134 pg/mL for p-tau217, 0.832 pg/mL for t-tau, 20.171 pg/mL for NfL and 142.515 pg/mL for GFAP.

Supplementary Table S9. Levels of blood biomarkers of Alzheimer's disease and hazard ratio (HR) of progression from normal cognition (NC) to mild cognitive impairment (MCI), reversion from MCI to NC, and progression from MCI to AD dementia, applying inverse probability weighting (IPW).

	**From NC to MCI**	**From MCI to NC**	**From MCI to AD dementia**
	HR (95%CI)	HR (95%CI)	HR (95%CI)
**Aß-42/40**			
Low vs High	0.88 (0.66, 1.16)	**0.66 (0.47, 0.92)**	**1.40 (1.09, 1.81)**
**P-tau181**			
High vs Low	1.00 (0.73, 1.36)	**0.59 (0.40, 0.87)**	**1.52 (1.18, 1.95)**
**P-tau217**			
High vs Low	1.21 (0.89, 1.65)	0.75 (0.50, 1.11)	**2.11 (1.62, 2.76)**
**T-tau**			
High vs Low	1.09 (0.82, 1.44)	0.98 (0.70, 1.38)	**1.44 (1.12, 1.84)**
**NfL**			
High vs Low	1.20 (0.87, 1.66)	0.72 (0.49, 1.06)	**2.36 (1.79, 3.11)**
**GFAP**			
High vs Low	1.13 (0.83, 1.53)	**0.67 (0.47, 0.97)**	**1.90 (1.44, 2.50)**

Hazard Ratios (HR) with 95% Confidence Intervals (CI) are derived from multistate Markov models, using age as time scale and adjusted for sex and education. Weights were derived from logistic regression models including age, sex, education, number of chronic diseases, and biomarker levels. Cut-offs: 0.057 for Aß-42/40 ratio, 1.512 pg/mL for p-tau181, 0.134 pg/mL for p-tau217, 0.832 pg/mL for t-tau, 20.171 pg/mL for NfL and 142.515 pg/mL for GFAP.

Supplementary Table S10. Number of elevated blood biomarkers of Alzheimer's disease (AD), among p-tau217, NfL and GFAP, and hazard ratio (HR) of progression from normal cognition (NC) to mild cognitive impairment (MCI), reversion from MCI to NC, and progression from MCI to all-cause dementia.

	**From NC to MCI**			**From MCI to NC**			**From MCI to all-cause dementia**		
	N transitions/ participants	HR (95%CI) Basic	HR (95%CI) Full adj.	N transitions/ participants	HR (95%CI) Basic	HR (95%CI) Full adj.	N transitions/ participants	HR (95%CI) Basic	HR (95%CI) Full adj.
**N of elevated biomarkers**									
None	148/832	Ref.	Ref.	143/832	Ref.	Ref.	39/832	Ref.	Ref.
Only one	81/449	1.15	1.25	80/449	0.85	0.95	58/449	**1.44**	1.41
		(0.74, 1.80)	(0.71, 2.17)		(0.50, 1.45)	(0.49, 1.84)		**(1.01, 2.06)**	(0.98, 2.02)
Only two	50/427	1.75	0.99	47/427	1.55	0.70	108/427	**2.74**	**2.36**
		(0.91, 3.38)	(0.52, 1.89)		(0.66, 3.65)	(0.30, 1.62)		**(1.87, 4.01)**	**(1.61, 3.46)**
All three	32/440	1.20	0.84	23/440	**0.34**	**0.26**	159/440	**2.58**	**2.22**
		(0.73, 1.96)	(0.44, 1.63)		**(0.17, 0.67)**	**(0.11, 0.61)**		**(1.80, 3.69)**	**(1.50, 3.28)**

Hazard Ratios (HR) with 95% Confidence Intervals (CI) are derived from multistate Markov models, using age as time scale. The basic model is adjusted for sex and education; the fully adjusted model is further adjusted for chronic kidney disease, heart diseases, cerebrovascular disease, anemia and obesity. Cut-offs: 0.134 pg/mL for p-tau217, 20.171 pg/mL for NfL and 142.515 pg/mL for GFAP. Abbreviations: MCI: mild cognitive impairment; NC: normal cognition; p-tau217: phosphorylated tau 217; NfL: neurofilament light chain; GFAP: glial fibrillary acidic protein.

Supplementary Table S11. Number of elevated blood biomarkers of Alzheimer's disease (AD), among p-tau217, NfL and GFAP, and hazard ratio (HR) of progression from normal cognition (NC) to mild cognitive impairment (MCI), reversion from MCI to NC, and progression from MCI to AD dementia.

	**From NC to MCI**			**From MCI to NC**			**From MCI to AD dementia**		
	N transitions/ participants	HR (95%CI) Basic	HR (95%CI) Full adj.	N transitions/ participants	HR (95%CI) Basic	HR (95%CI) Full adj.	N transitions/ participants	HR (95%CI) Basic	HR (95%CI) Full adj.
**N of elevated biomarkers**									
None	148/832	Ref.	Ref.	143/832	Ref.	Ref.	17/832	Ref.	Ref.
Only one	81/449	1.26	1.32	80/449	0.89	0.96	39/449	**2.08**	**1.98**
		(0.86, 1.86)	(0.87, 2.01)		(0.58, 1.38)	(0.59, 1.56)		**(1.27, 3.42)**	**(1.20, 3.26)**
Only two	50/427	1.56	1.48	47/427	1.19	1.07	57/427	**3.67**	**3.39**
		(0.95, 2.57)	(0.86, 2.55)		(0.65, 2.19)	(0.53, 2.18)		**(2.22, 6.04)**	**(2.04, 5.62)**
All three	32/440	1.53	1.52	23/440	**0.42**	**0.39**	99/440	**4.33**	**3.71**
		(0.99, 2.37)	(0.93, 2.48)		**(0.23, 0.75)**	**(0.20, 0.75)**		**(2.65, 7.06)**	**(2.22, 6.20)**

Hazard Ratios (HR) with 95% Confidence Intervals (CI) are derived from multistate Markov models, using age as time scale. The basic model is adjusted for sex and education; the fully adjusted model is further adjusted for chronic kidney disease, heart diseases, cerebrovascular disease, anemia and obesity. Cut-offs: 0.134 pg/mL for p-tau217, 20.171 pg/mL for NfL and 142.515 pg/mL for GFAP. Abbreviations: MCI: mild cognitive impairment; NC: normal cognition; p-tau217: phosphorylated tau 217; NfL: neurofilament light chain; GFAP: glial fibrillary acidic protein.

Supplementary Table S12. Combinations of p-tau217, NfL and GFAP and hazard ratio (HR) of progression from normal cognition (NC) to mild cognitive impairment (MCI), reversion from MCI to NC, and progression from MCI to all-cause dementia.

	**From NC to MCI**		**From MCI to NC**		**From MCI to all-cause dementia**	
	Basic HR (95%CI)	Fully adj HR (95%CI)	Basic HR (95%CI)	Fully adj HR (95%CI)	Basic HR (95%CI)	Fully adj HR (95%CI)
**P-tau217 and NfL**						
Low p-tau217 and Nfl	Ref.	Ref.	Ref.	Ref.	Ref.	Ref.
NfL high only	1.00 (0.61, 1.64)	0.85 (0.48, 1.51)	0.75 (0.39, 1.42)	0.59 (0.28, 1.25)	**1.69 (1.21, 2.35)**	**1.59 (1.13, 2.22)**
P-tau217 high only	3.35 (0.48, 23.24)	3.57 (0.45, 28.69)	3.50 (0.38, 31.76)	3.85 (0.37, 40.53)	**1.71 (1.12, 2.62)**	**1.71 (1.11, 2.63)**
High p-tau217 and NfL	1.24 (0.71, 2.14)	1.01 (0.52, 1.96)	0.63 (0.29, 1.34)	0.51 (0.20, 1.27)	**2.60 (1.88, 3.59)**	**2.29 (1.62, 3.24)**
**P-tau217 and GFAP**						
Low p-tau217 and GFAP	Ref.	Ref.	Ref.	Ref.	Ref.	Ref.
High GFAP only	1.32 (0.72, 2.41)	1.11 (0.64, 1.92)	1.14 (0.53, 2.46)	0.89 (0.44, 1.79)	**1.51 (1.07, 2.11)**	1.37 (0.98, 1.92)
High p-tau217 only	2.68 (0.94, 7.65)	2.08 (0.75, 5.71)	3.21 (0.93, 11.09)	2.44 (0.70, 8.47)	**2.09 (1.41, 3.10)**	**1.90 (1.28, 2.81)**
High p-tau217 and GFAP	1.05 (0.68, 1.64)	0.91 (0.54, 1.54)	**0.35 (0.19, 0.65)**	**0.33 (0.16, 0.66)**	**2.01 (1.49, 2.72)**	**1.77 (1.29, 2.44)**
**NfL and GFAP**						
Low NfL and GFAP	Ref.	Ref.	Ref.	Ref.	Ref.	Ref.
High GFAP only	1.15 (0.55, 2.41)	1.09 (0.54, 2.20)	0.79 (0.30, 2.07)	0.74 (0.31, 1.76)	**1.58 (1.08, 2.33)**	**1.57 (1.07, 2.31)**
High NfL only	1.19 (0.65, 2.18)	0.91 (0.47, 1.76)	0.92 (0.42, 1.99)	0.65 (0.29, 1.47)	**2.17 (1.50, 3.13)**	**1.99 (1.37, 2.87)**
High NfL and GFAP	0.96 (0.59, 1.58)	0.63 (0.36, 1.10)	**0.44 (0.24, 0.83)**	**0.25 (0.13, 0.51)**	**2.23 (1.63, 3.04)**	**1.91 (1.37, 2.66)**

Hazard Ratios (HR) with 95% Confidence Intervals (CI) are derived from multistate Markov models, using age as time scale. The basic model is adjusted for sex and education; the fully adjusted model is further adjusted for chronic kidney disease, heart diseases, cerebrovascular disease, anemia and obesity. Cut-offs: 0.134 pg/mL for p-tau217, 20.171 pg/mL for NfL and 142.515 pg/mL for GFAP. Abbreviations: MCI: mild cognitive impairment; NC: normal cognition; p-tau217: phosphorylated tau 217; t-tau: total tau; NfL: neurofilament light chain; GFAP: glial fibrillary acidic protein.

Supplementary Table S13. Combinations of p-tau217, NfL and GFAP and hazard ratio (HR) of progression from normal cognition (NC) to mild cognitive impairment (MCI), reversion from MCI to NC, and progression from MCI to Alzheimer's disease (AD) dementia.

	**From NC to MCI**		**From MCI to NC**		**From MCI to AD dementia**	
	Basic HR (95%CI)	Fully adj HR (95%CI)	Basic HR (95%CI)	Fully adj HR (95%CI)	Basic HR (95%CI)	Fully adj HR (95%CI)
**P-tau217 and NfL**						
Low p-tau217 and Nfl	Ref.	Ref.	Ref.	Ref.	Ref.	Ref.
NfL high only	1.20 (0.79, 1.83)	1.10 (0.69, 1.75)	0.91 (0.55, 1.51)	0.78 (0.44, 1.37)	**2.27 (1.50, 3.44)**	**2.09 (1.37, 3.17)**
P-tau217 high only	1.36 (0.78, 2.39)	1.63 (0.74, 3.56)	1.14 (0.57, 2.26)	1.49 (0.58, 3.83)	**2.06 (1.26, 3.38)**	**2.03 (1.23, 3.36)**
High p-tau217 and NfL	1.49 (0.99, 2.26)	1.33 (0.82, 2.15)	0.62 (0.37, 1.06)	**0.51 (0.28, 0.94)**	**3.62 (2.46, 5.32)**	**3.07 (2.04, 4.60)**
**P-tau217 and GFAP**						
Low p-tau217 and GFAP	Ref.	Ref.	Ref.	Ref.	Ref.	Ref.
High GFAP only	1.27 (0.81, 1.99)	1.28 (0.79, 2.06)	1.06 (0.63, 1.77)	1.05 (0.59, 1.88)	**1.81 (1.19, 2.77)**	**1.66 (1.09, 2.52)**
High p-tau217 only	1.57 (0.83, 2.97)	1.67 (0.79, 3.54)	1.52 (0.70, 3.29)	1.73 (0.69, 4.34)	**2.27 (1.41, 3.66)**	**2.09 (1.29, 3.37)**
High p-tau217 and GFAP	1.32 (0.89, 1.96)	1.37 (0.91, 2.079)	**0.46 (0.27, 0.77)**	**0.47 (0.26, 0.83)**	**2.97 (2.03, 4.36)**	**2.54 (1.72, 3.74)**
**NfL and GFAP**						
Low NfL and GFAP	Ref.	Ref.	Ref.	Ref.	Ref.	Ref.
High GFAP only	1.22 (0.74, 2.02)	1.25 (0.72, 2.18)	0.79 (0.43, 1.43)	0.82 (0.42, 1.57)	**1.98 (1.25, 3.13)**	**1.99 (1.26, 3.14)**
High NfL only	1.28 (0.79, 2.08)	1.22 (0.70, 2.12)	0.86 (0.48, 1.54)	0.79 (0.40, 1.55)	**2.71 (1.76, 4.17)**	**2.53 (1.64, 3.90)**
High NfL and GFAP	1.25 (0.85, 1.83)	1.07 (0.66, 1.74)	**0.58 (0.36, 0.92)**	**0.42 (0.23, 0.78)**	**3.18 (2.18, 4.65)**	**2.74 (1.84, 4.08)**

Hazard Ratios (HR) with 95% Confidence Intervals (CI) are derived from multistate Markov models, using age as time scale. The basic model is adjusted for sex and education; the fully adjusted model is further adjusted for chronic kidney disease, heart diseases, cerebrovascular disease, anemia and obesity. Cut-offs: 0.134 pg/mL for p-tau217, 20.171 pg/mL for NfL and 142.515 pg/mL for GFAP. Abbreviations: MCI: mild cognitive impairment; NC: normal cognition; p-tau217: phosphorylated tau 217; t-tau: total tau; NfL: neurofilament light chain; GFAP: glial fibrillary acidic protein.

Supplementary Table S14. Intra and inter assay precision expressed as coefficient of variation (% CV).

	Sample pool			Control 1			Control 2		
	**Conc**	**Within run**	**Between run**	**Conc**	**Within run**	**Between run**	**Conc**	**Within run**	**Between run**
**Aβ-40**	120.4	2.2	5.2	10.1	3.8	6.8	156.1	1.6	3.7
**Aβ-42**	6.6	4.8	7.4	3.4	6.1	8.2	76.8	2.1	4.7
**p-tau 181**	1.5	9.2	13.6	3.6	4.9	7.9	98.0	3.2	5.9
**p-tau 217**	0.14	13.3	17.6	0.8	8.8	12.0	7.7	6.1	11.2
**t-tau**	0.9	10.7	12.3	1.6	5.3	8.5	61.7	2.0	5.2
**NfL**	24.1	7.6	11.6	4.1	11.5	17.6	409.8	4.5	7.4
**GFAP**	194	13.1	21.4	89.8	11.4	17.4	10311	5.8	9.7

Conc: average of the concentrations (pg/mL) obtained in 39 runs.
Within run: CV (%) calculated on the triplicate pool, control 1 and control 2 included in each run (plate). The average CV for all runs is reported.
Between run: CV (%) calculated on the concentration values obtained for pool, control 1 and control 2 across all the runs (plate).

Supplementary Figure S1 Continuous values of blood biomarkers of Alzheimer's disease (AD) and hazard ratio (HR) of progression from normal cognition (NC) to mild cognitive impairment (MCI), reversion from MCI to NC, and progression from MCI to AD dementia.

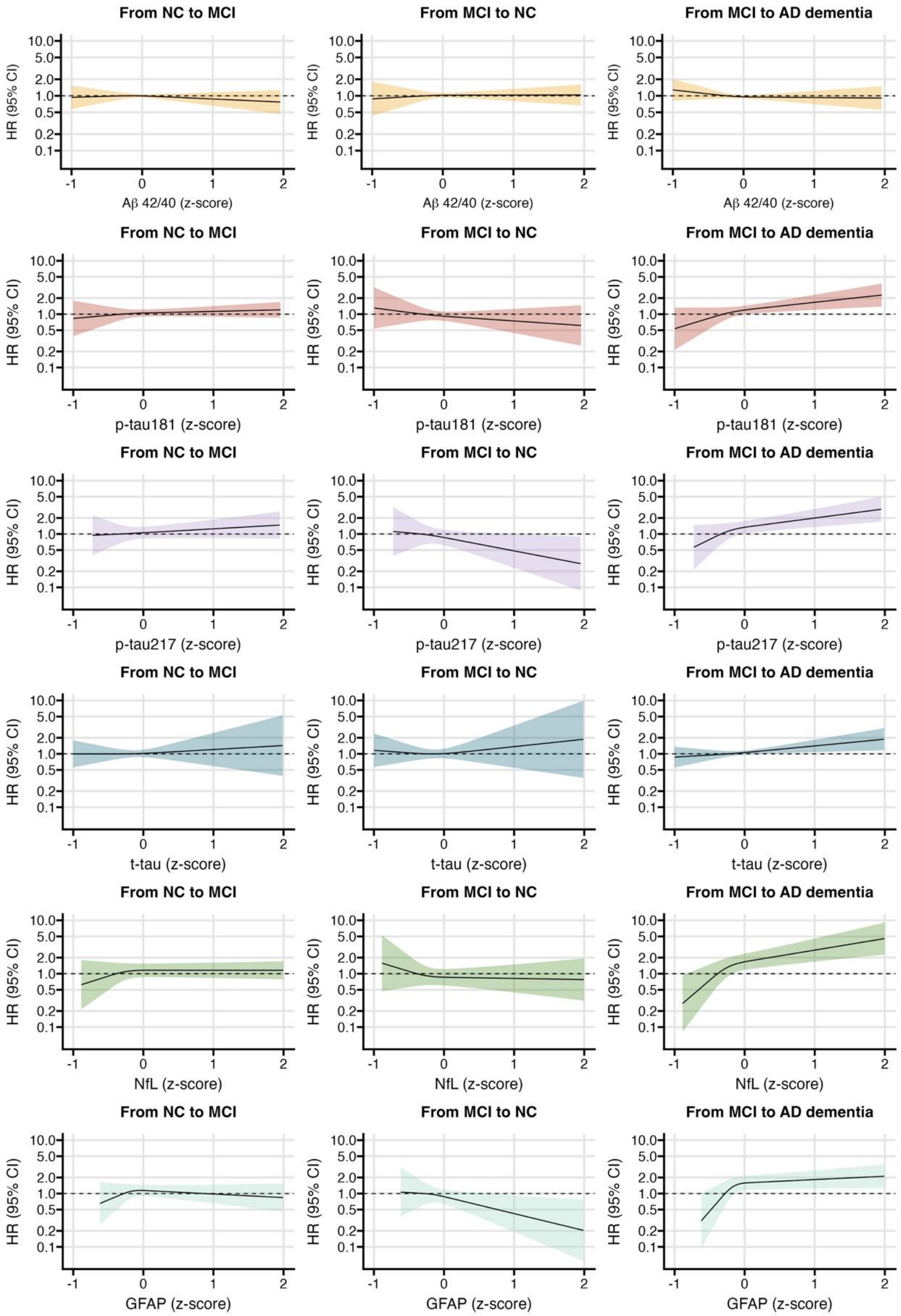

Hazard Ratios (HR) with 95% Confidence Intervals (CI) are derived from multistate Markov models, using age as time scale and adjusted for sex and education. AD blood biomarkers were converted into z-scores and modelled using restricted cubic splines with 3 prespecified knots at 25th, 50th, and 75th percentiles. The median value was chosen as reference for all the z-scored biomarkers. Abbreviations: Aß42/40: amyloid beta

42/40; MCI: mild cognitive impairment; NC: normal cognition; p-tau181: phosphorylated tau 181; p-tau217: phosphorylated tau 217; t-tau: total tau; NfL: neurofilament light chain; GFAP: glial fibrillary acidic protein. Colour schemes: Aß42/40: yellow; p-tau181: red; p-tau217: purple; t-tau: blue; NfL: green; GFAP: turquoise.

Supplementary Figure S2. Flow chart of study participation.

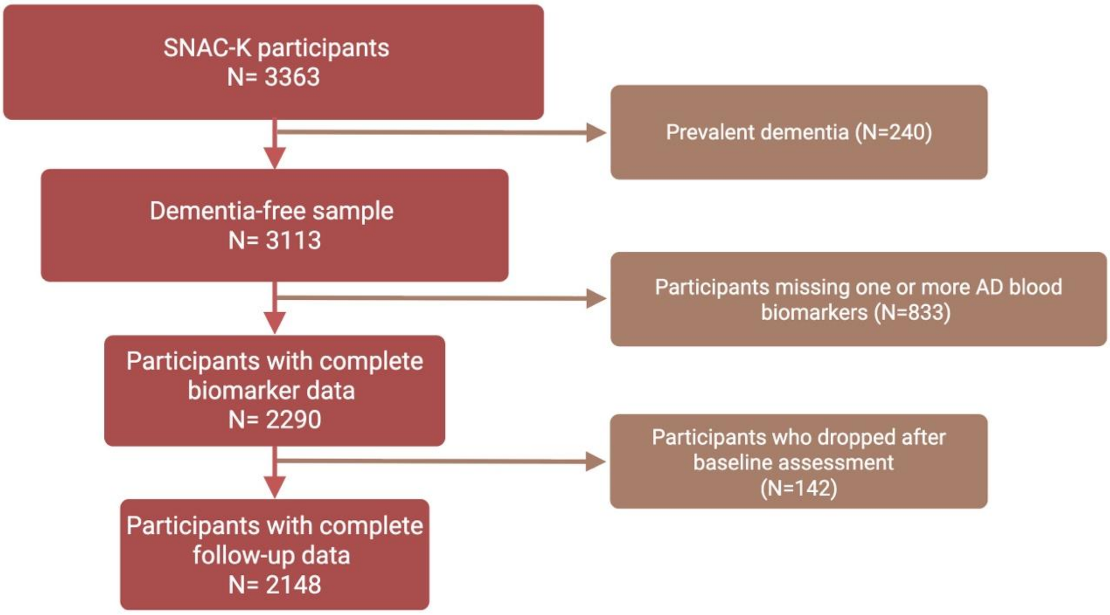

Created in BioRender. Valletta, M. (2026) https://BioRender.com/gqekh54

Supplementary Figure S3. Diagram of Multistate Markov models.

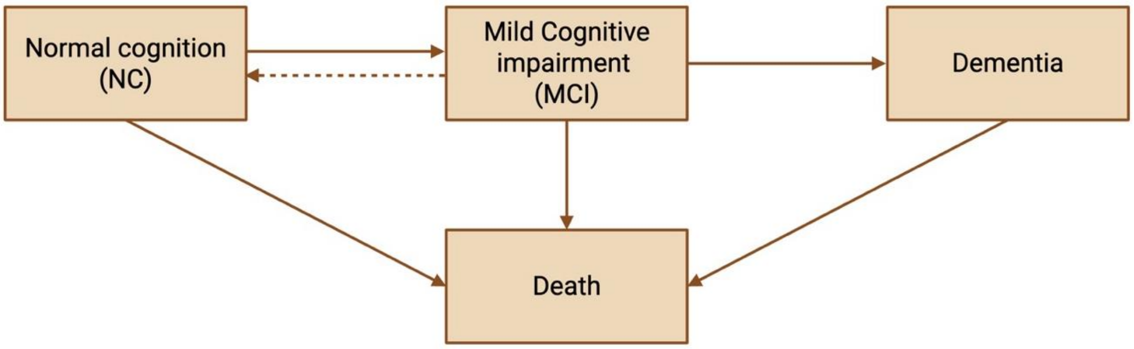

Created in BioRender. Valletta, M. (2025) https://BioRender.com/uwfngzg
