# Supplementary material for: Blood biomarkers of Alzheimer’s disease and progression across different stages of cognitive decline in the community
Source: Nat Commun. 2025 Nov 23;16:10412. doi: 10.1038/s41467-025-66728-2 (PMC12644782; doi:10.1038/s41467-025-66728-2)
Supplement: Supplementary file 2 — Reporting Summary [file 41467_2025_66728_MOESM2_ESM.pdf]

Corresponding author(s): Martina Valletta
Last updated by author(s): Nov 1, 2025

Reporting Summary

Nature Portfolio wishes to improve the reproducibility of the work that we publish. This form provides structure for consistency and transparency in reporting. For further information on Nature Portfolio policies, see our Editorial Policies and the Editorial Policy Checklist.

Statistics

For all statistical analyses, confirm that the following items are present in the figure legend, table legend, main text, or Methods section.

n/a	Confirmed	
☐	☒	The exact sample size (*n*) for each experimental group/condition, given as a discrete number and unit of measurement
☐	☒	A statement on whether measurements were taken from distinct samples or whether the same sample was measured repeatedly
☐	☒	The statistical test(s) used AND whether they are one- or two-sided<br>*Only common tests should be described solely by name; describe more complex techniques in the Methods section.*
☐	☒	A description of all covariates tested
☐	☒	A description of any assumptions or corrections, such as tests of normality and adjustment for multiple comparisons
☐	☒	A full description of the statistical parameters including central tendency (e.g. means) or other basic estimates (e.g. regression coefficient) AND variation (e.g. standard deviation) or associated estimates of uncertainty (e.g. confidence intervals)
☐	☒	For null hypothesis testing, the test statistic (e.g. *F*, *t*, *r*) with confidence intervals, effect sizes, degrees of freedom and *P* value noted<br>*Give P values as exact values whenever suitable.*
☒	☐	For Bayesian analysis, information on the choice of priors and Markov chain Monte Carlo settings
☒	☐	For hierarchical and complex designs, identification of the appropriate level for tests and full reporting of outcomes
☒	☐	Estimates of effect sizes (e.g. Cohen's *d*, Pearson's *r*), indicating how they were calculated

Our web collection on statistics for biologists contains articles on many of the points above.

Software and code

Policy information about availability of computer code

Data collection	Data were collected through dedicated interviews and forms (no software used). For biomarker analyses Quanterix SR-X software version 1.2.0 was used.
Data analysis	Statistical analyses were conducted using R version 4.3.1 (The R Foundation for Statistical Computing). To fit multistate models we used the msm package. The code for the analysis is available at https://github.com/ARCbiostat/biomdemstages/tree/main

For manuscripts utilizing custom algorithms or software that are central to the research but not yet described in published literature, software must be made available to editors and reviewers. We strongly encourage code deposition in a community repository (e.g. GitHub). See the Nature Portfolio guidelines for submitting code & software for further information.

Data

Policy information about availability of data

All manuscripts must include a data availability statement. This statement should provide the following information, where applicable:

- Accession codes, unique identifiers, or web links for publicly available datasets
- A description of any restrictions on data availability
- For clinical datasets or third party data, please ensure that the statement adheres to our policy

SNAC-K are sensitive data; thus, they cannot be shared publicly, but raw and analysed de-identified data can be requested by qualified researchers at https://www.snac-k.se/. The request will be reviewed by SNAC-K investigators to ensure confidentiality obbligations and intellectual property. A data sharing agreement must be signed prior to data release.

Research involving human participants, their data, or biological material

Policy information about studies with human participants or human data. See also policy information about sex, gender (identity/presentation), and sexual orientation and race, ethnicity and racism.

Reporting on sex and gender	Information concerning sex (biological attribute) is collected in SNAC-K, while gender is not. Analyses have been stratified by sex and reported in supplementary files.
Reporting on race, ethnicity, or other socially relevant groupings	SNAC-K participants are white 60+ individuals living in an affluent area of Stockholm; other ethnicities are not present in the sample.
Population characteristics	At each visit, participants underwent a standardized comprehensive evaluation by trained nurses, physicians, and psychologists. Clinical, laboratory, functional, and cognitive data were collected following standardized procedures. Participants were assessed at the research center or, if unable to travel, at home or institution. During nurses interview, information on demographics (age, sex, education) was obtained. Education was categorized into elementary, high school, and university or higher. To ensure a comprehensive assessment of participants health status, information on chronic diseases was collected by physicians through clinical evaluation, medical records, self-reports, laboratory tests, and medication use. Diseases were coded following the International Classification of Diseases 10th revision (ICD-10).<br>At baseline, median age of the 2148 study participants was 72.2 years, 61.5% were females, and 35.4% had a university level of education or higher.
Recruitment	The study population consists of community-dwelling and institutionalized individuals, aged 60 or older, living in the Kungsholmen district of Stockholm, Sweden. Between 2001 and 2004, a random sample of residents, stratified across 11 age cohorts, received a letter that invited them to participate in the baseline assessment. A total of 3363 individuals (response rate: 73.3%) were assessed at baseline, between 2001 and 2004. Those who did not participate were older and more likely females and institutionalized. Therefore, non-participants may have exhibited a worse global health status and a higher burden of chronic diseases.<br>Follow-ups were conducted every three years for participants ≥78 years and every six years for those <78 years.
Ethics oversight	The protocol for all waves of the SNAC-K study was approved by the Regional Ethical Review Board in Stockholm (Dnrs: KI 01-114, 04-929/3, Ö26-2007, 2009/595-32, 2010/447-31/2, 2013/828-31/3, 2016/730-31/1 and 2023-02375-02). Ethical standards of the declaration of Helsinki were followed throughout the study.

Note that full information on the approval of the study protocol must also be provided in the manuscript.

Field-specific reporting

Please select the one below that is the best fit for your research. If you are not sure, read the appropriate sections before making your selection.

- [x] Life sciences
- [ ] Behavioural & social sciences
- [ ] Ecological, evolutionary & environmental sciences

For a reference copy of the document with all sections, see nature.com/documents/nr-reporting-summary-flat.pdf

Life sciences study design

All studies must disclose on these points even when the disclosure is negative.

Sample size	No sample size calculations were conducted. The present study is based on the SNAC-K population-based cohort, which included 3363 individuals aged 60 years or older at baseline. For the statistical analyses presented in this manuscript, we used data from 2148 participants (see below).
Data exclusions	From the initial SNAC-K population, we selected dementia-free participants at baseline (n= 3123) and excluded individuals missing data on blood biomarkers (n= 833), obtaining a baseline population of 2290 participants. Participants with missing data on biomarkers were on average older, more frequently females, and had a lower educational level and a higher prevalence of chronic diseases than those with complete biomarker data. Overall, 142 (6.2%) participants dropped out, leaving a final analytical sample of 2148 participants with available follow-up data.
Replication	Several sensitivity analyses were performed. Models with age-dependent (i.e., ≤78 and >78 years old) hazard ratios for the biomarkers were fitted; the analyses were also stratified by sex. To assess whether including individuals with MCI at baseline could have influenced the results, the analyses were repeated excluding those with MCI at baseline. Sensitivity analyses using inverse probability weighting (IPW) to account for attrition yielded estimates that were consistent with the main results. When applying the cognitive impairment no dementia (CIND) operationalization instead of MCI, the results were comparable to those obtained with the MCI definition. Analyses were run multiple times to confirm the findings and data is available to qualified researchers to replicate (see data availability statement above).
Randomization	The current study is an observational study, so randomization was not applied.
Blinding	All biomarker analyses were performed by individuals who were blinded to the clinical data, and clinical diagnosis were made by physicians who were blinded to blood biomarkers results (i.e., they were made before the analyses of blood biomarkers).

Reporting for specific materials, systems and methods

We require information from authors about some types of materials, experimental systems and methods used in many studies. Here, indicate whether each material, system or method listed is relevant to your study. If you are not sure if a list item applies to your research, read the appropriate section before selecting a response.

Materials & experimental systems

n/a	Involved in the study
☐	☒ Antibodies
☒	☐ Eukaryotic cell lines
☒	☐ Palaeontology and archaeology
☒	☐ Animals and other organisms
☒	☐ Clinical data
☒	☐ Dual use research of concern
☒	☐ Plants

Methods

n/a	Involved in the study
☒	☐ ChIP-seq
☒	☐ Flow cytometry
☒	☐ MRI-based neuroimaging

Antibodies

Antibodies used

Simoa Neuro 3-plex A Kit was used to measure serum amyloid-β40 (Aβ40), amyloid-β42 (Aβ42) and total-tau (t-tau) (Quanterix, product number 101995 and Lot# 503659).
Simoa pTau-181 Advantage V2 Kit was used to measure serum phoshorylated-tau181 (p-tau181) (Quanterix, product number 103714 and Lot# 503703).
Simoa ALZpath p-Tau-217 Advantage PLUS Kit was used to measure serum phoshorylated-tau217 (p-tau217) (Quanterix, product number 104570 and Lot# 504307).
Simoa Neuro 2-plex B Kit was used to measure serum neurofilament light chain (NfL) and glial fibrillary acidic protein (GFAP) (Quanterix, product number 103520 and Lot# 503409).
For each kit, 25 µL of sample were diluted 1:4 and the assays were performed according to manufacturer instructions. The Quanterix instrument provides AEB (average enzyme per bead) values for calibrators, controls and samples. The Quanterix SR-X software automatically performs curve-fitting, extrapolation of concentrations and graphical representation using the calibrators and a four-parameter logistic (4PL) curve fit. Precision was estimated for each assay. Within run coefficient of variation (CV) was calculated on a triplicate serum pool (from SNAC-K samples), control 1 (Quanterix kit) and control 2 (Quanterix kit) included in each run (plate). The average CV for all runs is reported in supplementary material.

Validation

https://www.quanterix.com/wp-content/uploads/2022/10/pTau-181-Advantage-V2.1-HD-1-HD-X-Data-Sheet.pdf for p-tau181.
https://www.quanterix.com/wp-content/uploads/2020/12/N2PB_SR-X_Data_Sheet_rev01.pdf for NfL and GFAP
https://www.quanterix.com/wp-content/uploads/2020/12/Simoa_N3PA_Data_Sheet-SR-X_0.pdf for Aβ40, Aβ42 and t-tau
Validation of p-tau217 serum assay was carried out in a previous study conducted by our group in SNAC-K (DOI: 10.1038/s41591-025-03605-x).

Plants

Seed stocks

NA

Novel plant genotypes

NA

Authentication

NA
